# Quality of life and associated determinants of chronic pain among patients attending a primary healthcare clinic in Gqeberha: A cross-sectional study

**DOI:** 10.4102/safp.v68i1.6218

**Published:** 2026-02-12

**Authors:** Kemi D. Dele-Ijagbulu, Febisola I. Ajudua, Busisiwe Cawe

**Affiliations:** 1 Department of Family Medicine and Rural Health, Faculty of Health Sciences, Walter Sisulu University, Mthatha, South Africa

**Keywords:** chronic pain, quality of life, primary healthcare, pain management, South Africa

## Abstract

**Background:**

Chronic pain is a major global health challenge that impairs quality of life through physical disability, psychological distress, and socioeconomic burden. Despite its prevalence, limited research examines its multidimensional impact in South African primary healthcare. This study evaluated the quality of life and factors influencing chronic pain in patients attending a primary healthcare clinic in Gqeberha, South Africa.

**Methods:**

A cross-sectional study was conducted among 208 adults with chronic pain attending Walmer 14th Avenue Clinic. Data were collected using the Brief Pain Inventory, capturing demographics, pain severity, interference, relief, and management. Descriptive statistics, bivariate analysis, and multivariable logistic regression were performed using SPSS v29.

**Results:**

Participants had a mean age of 50.2 years; most were female (71.6%). Back pain was most common (43.8%), while pelvic/groin pain was most severe. Pain relief was inadequate in 74% of participants, with 15.9% reporting none. Sleep was the most affected quality-of-life domain (72% interference). Predictors of higher pain interference included pain severity (*p* < 0.001), pain relief (*p* = 0.003), marital status (*p* = 0.004), and employment status (*p* = 0.005). Disease-specific treatments and adjuvant therapies provided better relief than paracetamol, nonsteroidal anti-inflammatory drugs, or opioids.

**Conclusion:**

Severe pain and inadequate relief are prevalent in primary healthcare. Improving access to adjuvant and disease-specific therapies, as well as addressing socioeconomic factors, is thus essential for enhanced patient outcomes.

**Contribution:**

This study highlights the interplay between chronic pain, sociodemographic factors, and quality of life in South African primary care and underscores the need for tailored, multimodal, resource-sensitive pain management strategies to inform policy.

## Introduction

Chronic pain is a highly prevalent and debilitating condition that profoundly affects physical functioning, psychological well-being, and social relationships, thereby reducing overall quality of life.^1,2,3^ It contributes to functional impairment, mental health challenges, and substantial social and economic burdens.^4,5,6^ Globally, chronic pain affects approximately one in five adults, with prevalence estimates in South Africa ranging from 18.3% to 21%.^7,8,9,10^ Defined by the International Association for the Study of Pain as pain persisting for more than 3 months, it is among the most common reasons individuals seek medical care.^4,5,6^

Chronic pain interferes with multiple aspects of daily life, including sleep, mood, mobility, and interpersonal relationships.^7,8,9^ Affected individuals often experience reduced mobility, social isolation, decreased productivity, financial strain, and heightened risk of mental health disorders such as depression, anxiety, and sleep disturbances.^10,11,12^ Its consequences extend beyond the individual, placing strain on families, workplaces, and health systems.^2,13,14^ Chronic pain is also a leading cause of workplace absenteeism and escalating healthcare costs.^14,15,16^

In primary healthcare settings, where most patients with chronic pain seek treatment, chronic pain remains under-researched, poorly understood and undertreated.^17,18,19^ Pain management in these contexts is often suboptimal, constrained by limited resources, insufficient pain assessment, and the absence of integrated, multimodal pain management approaches.^20,21,22^ Primary healthcare clinics are frequently ill-equipped to address the complex and multidimensional nature of chronic pain, relying on general pain relief strategies rather than evidence-based, tailored interventions.^23,24^

This study highlights the need for a holistic, patient-centred approach to chronic pain management in South African primary healthcare, with an emphasis on improving pain relief strategies to enhance patients’ quality of life. The theoretical framework is grounded in the biopsychosocial model of chronic pain, which recognises pain as more than a physical symptom, but also as an experience shaped by psychological, social, and environmental factors.^25,26,27^ All pains are not the same; therefore, viewing chronic pain as a single homogeneous entity is not helpful.^5,28^ The biopsychosocial model thus highlights three presumed pathophysiological pathways to pain, including the nociceptive, neuropathic, and nociplastic pathways.^5,6^ It further integrates biological factors (such as pain severity, associated symptoms, comorbidities, and medication use), psychological factors (including depression, anxiety, and sleep disturbances), and social determinants (such as socioeconomic status, employment, and family support).^3,29^ Together, these elements form the basis for developing individualised, comprehensive management plans for patients with chronic pain.^3,29^

While chronic pain has been extensively studied in the context of specific diseases, a critical gap in understanding its broader effects on patients’ quality of life within primary healthcare contexts still exists.^22,30,31^ Most existing research focuses on the prevalence of chronic pain, its association with specific medical conditions, or pharmacological interventions, with limited emphasis on how chronic pain affects the overall quality of life of primary healthcare patients.^29,32,33^ Evidence is particularly scarce on the socio-demographic and psychosocial determinants of pain-related disability in resource-limited settings, where access to specialised pain management services is restricted.^34,35^ In South Africa, studies have predominantly examined chronic pain in the context of hospital-based or disease-specific populations, offering little insight into how chronic pain influences multiple quality-of-life domains in primary healthcare.^29,32,33^

This study sought to bridge this gap by evaluating the quality of life of patients with chronic pain attending a primary healthcare clinic in Gqeberha, South Africa. We evaluated pain characteristics, including location, duration, severity, and self-reported management strategies, to assess how chronic pain interferes with multiple quality-of-life domains. In addition, we investigated key socio-demographic and clinical determinants of chronic pain and their contribution to pain-related disability within this primary healthcare population.

## Research methods and design

### Study design

This was a cross-sectional, quantitative study designed to evaluate the quality of life of patients with chronic pain attending a primary healthcare clinic in Gqeberha, South Africa.

### Setting

The study was conducted at Walmer 14th Avenue Clinic, a government-funded primary healthcare facility located in Gqeberha, Eastern Cape, South Africa. Gqeberha, an urban centre within the Nelson Mandela Bay Metropolitan Municipality, has a population of approximately 1.2 million and is served by 44 primary care facilities, including clinics and community health centres.^36,37^ Walmer 14th Avenue Clinic manages an estimated 4000 patient visits per month and offers a comprehensive range of primary care services. These include general medical consultations, chronic disease management, maternal and child health, mental healthcare, infectious disease management, and human immunodeficiency virus (HIV) and acquired immunodeficiency syndrome (AIDS) services. The clinic primarily serves a diverse, low-income urban population with limited access to specialised pain management services. The predominant languages spoken are English, isiXhosa, and Afrikaans. Care is delivered by a multidisciplinary team comprising family medicine registrars, medical officers, primary healthcare nurses, community health workers, and allied health professionals. Patients requiring higher levels of care are referred to regional and tertiary hospitals within the metropolitan area.

### Study population and sampling strategy

The study population included adult patients (≥ 18 years) with chronic pain who attended the clinic for routine care during the study period. Chronic pain was defined, in line with the International Association for the Study of Pain, as persistent, unresolved, or recurrent pain lasting 3 months or longer.^4,5^ Patients with acute pain conditions, cognitive impairment that limited consent or questionnaire completion, unwillingness to participate, or refusal to provide informed consent were excluded.

### Sampling method

A systematic sampling method was used to recruit participants. Every third eligible patient in the clinic’s waiting area during the data collection period was invited to participate. Initial screening involved chronicity questions and clinical history review; the researcher asked if the potential participants had experienced any pain lasting at least 3 months. Patients reporting pain for less than 3 months were excluded before completing the full questionnaire. If a patient declined participation, recruitment continued with the next patient until the predetermined sample size was achieved. The sample size was calculated using Cochran’s formula for cross-sectional studies, based on a 95% confidence level, a 5% margin of error, and an estimated prevalence of chronic pain in the population based on recent studies.^31,38^ This yielded a minimum required sample of 196 participants. Ultimately, 208 patients were enrolled in the study.

### Data collection

Quantitative data were collected between February and April 2024 using a structured questionnaire incorporating the long-form Brief Pain Inventory (BPI). The instrument was used without modification for the pain severity, interference, and relief domains. The BPI has been validated in both South African and international settings, and it demonstrates high internal consistency (Cronbach’s α > 0.85).^9,10^ The questionnaire comprised three sections: (1) identification of pain sites and ratings of pain severity; (2) self-reported treatments and perceived relief; and (3) assessment of pain interference with quality of life. Pain severity, interference, and relief were measured across four time points (pain severity as worst pain, least pain, average pain, and current pain) using a 0–10 numerical rating scale where 0 is no pain, and 10 is the worst imaginable pain. Pain interference was also assessed across seven domains: walking, general activity, work, mood, sleep, relationships, and enjoyment of life. These were subjective, based on the participants’ experiences. A 0–10 numerical rating scale was also used, where 0 is no interference, and 10 is the worst imaginable interference. Pain relief was, however, measured in percentages, ranging from 0% pain relief to 100% pain relief. Additional data collected included sociodemographic characteristics, pain duration, anatomical pain sites, comorbidities, and pain management strategies.^30,31^

### Pilot study

A pilot study was conducted on 20 participants at the Family Medicine Department outpatient clinic in Gqeberha to assess the clarity and suitability of the questionnaire used in this research on a comparable population. The questionnaires were presented in English, isiXhosa, and Afrikaans, based on the preferences. Feedback from the pilot confirmed that the instrument was unambiguous and appropriate for addressing the study objectives. Data from the pilot study were excluded from the final analysis.

### Data analysis

Quantitative data were captured electronically using Google Sheets and subsequently exported to the IBM Statistical Package for Social Sciences software version 29.0 (IBM SPSS, Chicago, Illinois, United States) for analysis. The data were checked for accuracy, completeness, and consistency, and then cleaned and coded. To maintain anonymity, each participant was assigned a unique study identifier. The dataset was confirmed to be normally distributed.

For analysis, scores on the numerical rating scale were categorised as follows: pain severity into mild (1–3), moderate (4–6), and severe (7–10); pain relief into no relief (0% – 9%), poor relief (10% – 39%), moderate relief (40% – 69%), and good relief (70% – 100%); and pain interference into no interference (0), mild (1–3), moderate (4–6), and severe (7–10). Pain duration was grouped into < 1 year, 1–5 years, 5–10 years, and > 10 years. Anatomical pain sites were classified into nine categories: lower limbs, upper limbs, headache/facial pain, neck/shoulder, backache, abdomen, chest, pelvis, and generalised joint pain.

Self-reported pain management strategies were grouped into 12 categories: (1) no treatment; (2) paracetamol only; (3) nonsteroidal anti-inflammatory drugs (NSAIDs) only; (4) mild opioids only; (5) paracetamol and NSAIDs; (6) paracetamol and mild opioids; (7) NSAIDs and mild opioids; (8) paracetamol, NSAIDs, and mild opioids; (9) adjuvant medications (e.g. amitriptyline, orphenadrine, prednisone, pregabalin, carbamazepine, lidocaine patches, cannabis); (10) disease-specific modalities (e.g. antihypertensives, nitrates, disease-modifying antirheumatic drugs, asthma kits, gout kits, migraine kits); (11) physical therapies (e.g. massage, heat/cold rubs, gym, exercise, braces, orthotics); and (12) water only. These categories were created based on the responses given by the participants

Descriptive statistics were used to summarise data and were presented in tables and graphs. Numerical variables (e.g. age, pain severity, pain relief, pain interference, and domain-specific interference) were expressed as means with standard deviations (s.d.), while categorical variables (e.g. gender, population group, employment, and marital status) were presented as frequencies and percentages.

Inferential analyses examined associations between variables and identified predictors of pain severity, pain interference, and treatment outcomes. Bivariate associations were assessed using Chi-square tests and Pearson’s correlation to determine significant associations and the direction and strength of such associations. Chi-square tests were used for categorical associations, Pearson’s correlation for numerical variables, and significant variables were entered into multivariable logistic regression. Significant variables were included in multivariable logistic regression models to identify predictors of quality-of-life interference, and the corresponding odds ratios were reported. All reported *p*-values were two-tailed. Associations were considered statistically significant if the *p*-value was < 0.05 at a 95% confidence interval (CI).

### Ethical considerations

Ethical approval for this study was obtained from the Walter Sisulu University Research Ethics & Biosafety Committee on 02 August 2023 with ethical clearance number 072/2022. Permission to conduct the research was also granted by the Eastern Cape Department of Health through the National Health Research Database (reference Number: EC_202311_017), the Office of Clinical Governance of the Nelson Mandela Bay Health District, and the facility manager of Walmer 14th Avenue Clinic. Written informed consent was obtained from all participants. Privacy and confidentiality were strictly maintained; data were anonymised using unique identifiers and stored securely in a password-protected database.

## Results

### Baseline characteristics

A total of 208 participants met the eligibility criteria and were included in the analysis. Their baseline characteristics are summarised in Table 1. The mean age was 50.2 (s.d. ± 14.99) years. The majority were female (71.6%) and of black African descent (72.1%). Most participants had completed high school (68.3%), and just over half were employed (53.4%). Hypertension was the most common comorbidity (51.4%), followed by HIV (21.6%) and arthritis (15.9%).

**TABLE 1:** Baseline characteristics of study participants (*N* = 208).

Variables	Characteristics	*n*	%
Age (years)	18–30	25	12.0
	31–45	60	28.8
	46–60	76	36.5
	61–75	34	16.3
	> 75	13	6.3
Gender	Male	59	28.4
	Female	149	71.6
Population group	Black people	150	72.1
	White people	36	17.3
	Coloured people	22	10.6
Level of education	None	2	1.0
	Primary	25	12.0
	Secondary	142	68.3
	Tertiary	39	18.7
Marital status	Single	98	47.1
	Married	84	40.4
	Divorced	16	7.7
	Widowed	10	4.8
Employment status	Unemployed	50	24.0
	Employed	111	53.4
	Pensioner	47	22.6
Pain duration	3–12 months	62	29.8
	1–5 years	96	46.1
	5–10 years	18	8.7
	> 10 years	32	15.4
Comorbid condition	None	43	20.7
	HPT	107	51.4
	HIV	45	21.6
	Arthritis	33	15.9
	DM	32	15.4

*N*, total number of participants; HPT, hypertension; HIV, human immunodeficiency virus; DM, diabetes mellitus.

### Chronic pain characteristics

The mean duration of chronic pain was 5.8 years (s.d. ± 4.3), with 1–5 years being the most frequently reported duration (46.1%), and 15.4% of participants experiencing pain for more than 10 years (Table 1). Anatomical pain sites and their associations with pain severity and interference are presented in Table 2. The most reported site was the back (43.8%), followed by the lower limbs (36.1%) and headache/facial pain (26.4%). Although pelvic and groin pain accounted for only 9.1% of cases, it was reported as the most severe pain location (*p* < 0.001). Chest pain was 2.3 times more prevalent among females than males (*p* = 0.014). Back pain and pelvic pain showed significant associations with pain severity (*p* = 0.045 and *p* < 0.001, respectively), while lower limb, joint, and chest pain were most strongly associated with quality-of-life interference (*p* = 0.002, *p* = 0.016, and *p* = 0.005, respectively).

**TABLE 2:** Anatomical sites of chronic pain and their association with pain severity and interference (*N* = 208).

Anatomical site†	Participants		Pain severity		Pain interference	
	*n*	%	*χ* ^2^	*p*-value	*χ* ^2^	*p*-value
Backache	91	43.8	6.200	0.045	2.07	0.558
Lower limb	75	36.1	3.630	0.163	15.14	0.002
Headache or face	55	26.4	1.260	0.533	6.73	0.081
Joints	39	18.8	4.550	0.103	10.31	0.016
Upper limb	35	16.8	2.570	0.276	5.56	0.135
Abdomen	34	16.3	0.020	0.988	6.53	0.088
Neck or shoulder	31	16.3	2.280	0.320	1.32	0.732
Chest	27	13.0	1.193	0.384	12.66	0.005
Pelvic or groin	19	9.1	14.160	< 0.001	1.99	0.574

Note: Significant if *p* < 0.05.

*χ*^2^, Chi-square (test of significance for categorical data).

† , Participants could have more than one pain site.

### Chronic pain severity

**FIGURE 1:**
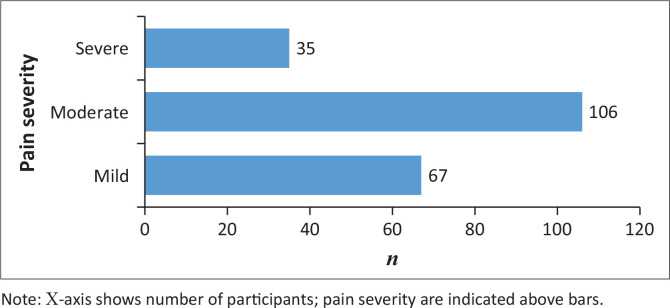
Distribution of chronic pain severity among participants.

Pain severity, assessed on a 10-point scale, revealed that 51.0% of participants reported moderate pain (4–6/10), while 16.8% reported severe pain (≥ 7/10) (see Figure 1). The mean pain severity score was 4.67 (s.d. ± 1.94), reflecting moderate overall severity. Pain scores were higher among females than males, although this difference was not statistically significant (*p* = 0.119). The age distribution exhibited a bimodal peak in pain interference; pain was more severe in the younger participants (< 30 years) and older participants (> 75 years). In addition, most chronic pain patients also had medical comorbidities, with only one-fifth (20.7%) of participants reporting no medical comorbidities (Table 1). Despite the high rates of comorbidities, there was no significant relationship observed between chronic pain severity and the presence of chronic diseases (*p* = 0.091).

### Pain management and relief

The participants reported a mean pain relief score of 39.86% (s.d. ± 29.37), indicating mild to moderate pain relief. About 74.0% of participants reported inadequate pain relief, with 15.9% experiencing no relief at all. Table 3 reveals the relationship between the treatment modalities and pain relief. Paracetamol was the most used analgesic, with 29.7% of users reporting good relief (*p* = 0.034). Disease-specific treatments such as antihypertensives, isosorbide dinitrate, metered dose inhalers, migraine kits, and disease-modifying antirheumatic drugs provided the highest reported pain relief (42.9%, *p* = 0.002). Likewise, adjuvant pain therapies, physiotherapy and cognitive behavioural therapy were used by a minority of participants but demonstrated statistically significant pain relief (31.6%, *p* = 0.042). Notably, opioid use did not show a significant association with pain relief (*p* = 0.095).

**TABLE 3:** The treatment modalities and a multivariable logistic regression model for the association between self-reported pain treatment and chronic pain relief (*N* = 208).

Pain treatment	Participants		% Good relief	OR†	*p*-value
	*n*	%			
Nil	19	9.1	0.0	1.00	-
Water	12	5.8	0.0	1.07	0.902
Paracetamol only	37	17.8	29.0	2.43	0.034
NSAID only	25	12.0	28.0	2.40	0.051
Opioids only	11	5.3	9.1	1.91	0.237
Paracetamol and NSAIDs	9	4.3	33.3	0.53	0.309
Paracetamol and opioid	22	10.6	36.4	0.89	0.802
NSAID and opioid	8	3.8	37.5	2.49	0.130
Paracetamol NSAID opioid	21	10.1	33.3	2.38	0.061
Adjuvant medications	19	9.1	31.6	2.62	0.042
Physical therapies	11	5.3	18.2	1.91	0.237
Disease-specific treatment	14	6.7	42.9	4.85	0.002

† , OR, odds ratio: measure of association between an exposure and an outcome; NSAID, nonsteroidal anti-inflammatory drug.

### Interference with quality of life

The mean overall pain interference score was 3.43/10 (s.d. ± 1.99), reflecting mild-to-moderate disruption in quality of life. Table 4 summarises the interference of chronic pain on the quality-of-life domains. General activity was substantially affected, with a mean score of 4.01 (s.d. ± 3.17) and 86% of participants reporting interference. Sleep emerged as the most affected domain with a mean score of 4.60 (s.d. ± 3.62); 72% of participants reported sleep disturbances, including 39.3% who experienced severe interference. Mood disturbances were also common, with one-quarter of participants reporting severe impairment. In contrast, interpersonal relationships were the least affected, with only 42% of participants reporting disruptions in social interactions. Overall, chronic pain interference with quality of life was statistically significant at *p* < 0.001.

**TABLE 4:** Chronic pain interference with quality of life (*N* = 208).

Quality of life	Mean	s.d.	% No interference	% All interference	% Severe interference
All subdimensions	3.43	1.9	10.6	89.4	6.3
Walking	3.19	3.1	29.3	70.4	22.6
Activity	4.01	3.2	13.9	86.1	30.3
Work	3.77	3.0	20.2	79.8	25.5
Relationship	1.58	2.5	56.7	43.3	8.7
Enjoyment	3.36	3.2	29.8	70.3	21.6
Mood	3.49	3.1	23.6	76.4	24.0
Sleep	4.60	3.6	27.9	72.1	39.3

s.d., standard deviation.

### Bivariate associations between predictor variables and pain interference subdimensions

Bivariate analysis using the Chi-square Test of Independence (Table 5) revealed several significant associations (*p* < 0.05) between sociodemographic/clinical variables and pain interference subdimensions. Population group, marital status, employment status, duration of pain, relief from treatment, and pain severity showed multiple significant relationships with pain interference outcomes. Marital status was significantly associated with overall pain interference (*p* = 0.004), and employment status was linked to both overall interference (*p* = 0.005) and interference with walking (*p* < 0.001). Pain severity was significantly related to all subdimensions except relationship (*p* = 0.290), while duration of pain was significantly associated with interference in daily activities (*p* = 0.002) and interpersonal relationships (*p* = 0.004). These results indicate that both sociodemographic and clinical characteristics influence the extent to which chronic pain affects different areas of patients’ lives.

**TABLE 5:** A Chi-square test of independence to determine the bivariate associations between the predictor variables and pain interference subdimensions.

Interference	Age	Gender	Popn	Educ	Mar	Empl	Dur	Relief	Severe
All	0.66	0.37	0.040	0.51	0.00	0.00	0.44	0.00	< 0.00
Walking	0.07	0.44	0.042	0.57	0.20	< 0.00	0.04	0.05	0.00
Activity	0.02	0.06	0.205	0.15	0.05	0.61	0.00	0.30	0.00
Work	0.49	< 0.00	0.075	0.69	0.02	0.06	0.10	0.01	< 0.00
Relationship	0.15	0.44	0.158	0.18	0.04	0.02	0.00	0.23	0.29
Enjoyment	0.07	0.51	0.004	0.07	0.56	0.00	0.34	0.03	< 0.00
Mood	0.46	0.49	0.270	0.07	0.02	0.08	0.29	0.19	0.00
Sleep	0.26	0.96	0.438	0.012	0.72	0.08	0.02	0.03	0.09

Popn, population group; Educ, education; Mar, marital status; Empl, employment status; Dur, pain duration; Relief, pain relief; Severe, pain severity.

### Predictors of pain interference

Multivariable logistic regression analyses were performed to identify the key predictors of chronic pain severity, pain relief, and pain interference, as presented in Table 6. Pain severity emerged as the most significant predictor of adverse quality-of-life outcomes (*p* < 0.001). Participants who reported insufficient pain relief also experienced significantly greater pain interference in daily activities (*p* = 0.003), thus underscoring the urgent need for more effective pain management strategies. The duration of pain, however, was not a significant predictor of pain interference (*p* = 0.087), suggesting that chronic pain-related disability may be more closely linked to severity and management rather than the length of time an individual has been in pain. Furthermore, sociodemographic and economic factors were also associated with pain outcomes. Unmarried individuals experienced significantly worse pain-related quality-of-life interference (*p* = 0.004), while unemployed participants also reported significantly higher pain interference scores (*p* = 0.005). Racial disparities were observed, with black African participants reporting higher pain interference than participants from other racial groups (*p* = 0.040).

**TABLE 6:** Predictors of pain interference.

Variable	Pain severity		Pain relief		Pain interference	
	*χ* ^2^	*p*-value	*χ* ^2^	*p*-value	*χ* ^2^	*p*-value
Age	8.59	0.378	24.29	0.019	9.48	0.661
Gender	4.10	0.129	5.26	0.154	3.12	0.374
Population group	2.94	0.567	4.33	0.632	13.19	0.040
Education	10.41	0.108	7.89	0.545	8.25	0.510
Marital status	17.24	0.008	16.24	0.062	24.52	0.004
Employment status	0.61	0.962	9.97	0.126	18.42	0.005
Pain duration	20.53	0.002	19.03	0.025	8.93	0.443
Pain treatment	35.86	0.031	127.63	< 0.001	46.84	0.056
Pain relief	32.24	< 0.001	-	-	24.59	0.003
Pain severity	-	-	33.24	< 0.001	34.65	< 0.001

Note: Significant if *p* < 0.05.

*χ*^2^, Chi-square (test of significance for categorical data).

## Discussion

This study evaluated the quality of life of patients with chronic pain attending a primary healthcare clinic in Gqeberha, South Africa. We identified a high prevalence of moderate-to-severe pain, significant interference across multiple quality-of-life domains, and poor levels of pain relief. Multivariable analyses highlighted pain severity, inadequate pain relief, unemployment, and unmarried status as key predictors of pain interference, while racial disparities were also observed. These findings underscore the urgent need for more effective and contextually appropriate pain management strategies within primary healthcare.

This study assessed the multidimensional nature of chronic pain using the Brief Pain Inventory, an approach consistent with other research that has applied multidimensional tools to evaluate pain determinants.^29,31,39,40^ The mean age of participants was 50.2 years, supporting prior evidence that chronic pain is more common among older adults.^27,31,38^

Consistent with global and South African research, back pain emerged as the most common pain site and was strongly associated with disability (43.8%), followed by headaches (26.4%) and knee pain (23.1%). These findings align with Mills, Nicolson, and Smith,^28^ who reported that back pain is the leading chronic pain worldwide, and Haraldstad et al.^29^ emphasised the dominance of back pain and its strong correlation with disability, as well as occupational, musculoskeletal, and lifestyle links.

Similarly, this study found that females had a 25% higher likelihood than males of experiencing chronic pain interference. This is consistent with previous research showing that women with chronic pain often report poorer quality of life compared to men.^27,31,38^ This disparity may be explained by a combination of hormonal, psychosocial, and cultural factors that shape women’s pain experiences and influence their healthcare-seeking behaviours,, as suggested by Pandelani et al.^31^ in their research. Importantly, the regression model revealed bimodal peaks in pain interference among participants younger than 30 years and those older than 75 years. suggesting that pain is not only age-related but may also be shaped by sociodemographic factors, with younger adults potentially facing unique psychological vulnerabilities and health system barriers than in older adults.^31^ While many studies have documented the association between advancing age and chronic pain, limited research has examined the burden of pain interference among younger populations. Future studies are necessary to explore these early-onset patterns and their underlying mechanisms.

Pain management in this population was suboptimal. Paracetamol (52.3%) and NSAIDs (47.8%) were the most used medications, yet both provided only modest relief, while opioids (41.5%) demonstrated limited effectiveness. This finding supports the work of Cohen, Vase, and Hooten,^41^ who argued that long-term opioid use offers little benefit and carries significant risks of dependence and harm.^41^ In contrast, non-pharmacological treatments such as physiotherapy and psychological support, disease-specific treatments and adjuvant modalities were associated with superior outcomes. This aligns with Borrelli and Bettinger,^17^ who highlighted the importance of multimodal pain management in primary healthcare settings. However, the unexpectedly low effectiveness of NSAIDs (47.8%) contradicts previous reports by Gebke et al.,^21^ which indicated that NSAIDs provide 60% to 70% relief in similar populations. This discrepancy may be attributed to differences in patient adherence, comorbidities, or access to care, underscoring the need to adapt evidence-based strategies to the realities of resource-limited primary healthcare settings.

Chronic pain substantially interfered with multiple quality-of-life domains in this study, with 72% of participants reporting sleep disturbances, 22% experiencing severe mobility problems, 24% experiencing severe mood disturbances, and 25% facing severe work-related impairment. These findings are consistent with international evidence linking chronic pain to insomnia, depression, and reduced productivity.^3,7,35^ According to Sun et al.,^7^ over 70% of chronic pain patients experience disrupted sleep because of pain-related discomfort and nighttime awakenings. Sleep disruption creates a vicious cycle in which inadequate rest exacerbates pain perception and lowers tolerance, further intensifying the overall burden of pain. Philpot, Schumann, and Ebbert,^35^ in their report, also indicated that chronic pain is a major contributor to work absenteeism and reduced productivity. However, the relatively lower prevalence of social withdrawal, with only 8.7% experiencing severe relationship interference, contradicts the findings of Hadi, McHugh, and Closs^3^ who reported that over 50% of chronic pain patients experience significant social disengagement. This discrepancy may reflect the protective effects of strong family and community support systems in South Africa.

Patients with chronic pain also experienced a substantial psychological burden, with 25% reporting symptoms of severe mood disorder. This finding is consistent with the findings of Pereira et al.,^39^ who reported that chronic pain increases the risk of mental health disorders, primarily because of the prolonged distress, disability, and social isolation associated with the condition.^39^ However, the severe mood disorder rates observed in this study were lower than those reported by van Vreede, Parker, and van Nugteren,^40^ who found that over 40% of chronic pain patients develop clinical depression. This difference may be explained by the absence of standardised mental health assessment tools, such as the PHQ-9, which may have led to underestimation of psychological distress.

A key component of this study was identifying factors associated with higher pain burden and reduced pain relief. Pain severity was the strongest predictor of pain interference (*p* < 0.001), with higher pain levels directly correlating with poorer quality-of-life outcomes. This supports the findings by Haraldstad et al.^29^ that pain severity is the most critical determinant of quality of life interference and disability. Other significant predictors included unemployment (*p* = 0.005), marital status (*p* = 0.008), and inadequate pain relief (*p* < 0.001). The association between employment status and increased pain burden further supports Pandelani et al.^31^ who reported that financial instability and joblessness exacerbate chronic pain by limiting healthcare access and increasing psychosocial stress.^31^ Additionally, marital status played a role, with unmarried participants reporting greater pain interference. This aligns with Miaskowski et al.^25^ who suggested that social support from spouses or partners serves as a protective factor, enhancing pain coping mechanisms.^25^

This study did not identify any significant gender-based differences in pain burden, which contradicts the findings of Mills, Nicolson, and Smith^28^ who reported that women typically experience greater pain interference than men because of biological and hormonal factors. The absence of gender disparities in the present study may reflect differences in self-reported pain perception, limitations in sample size, or cultural factors influencing how pain is expressed. Similarly, the racial disparities identified (*p* = 0.040) were weaker than those reported by Kamerman et al.^38^ who reported that racial and socioeconomic inequalities significantly influence the chronic pain burden in South Africa, primarily because of unequal access to healthcare resources. Uniform accessibility of primary healthcare services to the different population groups within the clinic setting may have mitigated broader disparities, thereby accounting for the weaker association in this study.

### Strengths and limitations

This study employed the Brief Pain Inventory, a validated multidimensional tool, to quantitatively assess quality of life among patients with chronic pain. The use of a 24 h recall method minimised recall bias and strengthened the validity of the findings. Furthermore, the inclusion of multivariable logistic regression enhanced the robustness of the analysis by identifying independent predictors of pain interference and pain-related disability. However, certain limitations must be acknowledged. Firstly, the study was conducted at a single primary healthcare clinic, which restricts the generalisability of the findings to other settings. Secondly, the cross-sectional design was utilised; hence, causality could not be inferred. Thirdly, reliance on self-reported measures introduces potential reporting bias, given the subjective nature of pain. Despite these limitations, the findings remain highly relevant for healthcare providers and policymakers working to improve pain management in resource-limited settings.

### Implications of the study

The findings of this study demonstrate that pain severity, inadequate pain relief, and sociodemographic factors such as employment status and marital status are significant determinants of quality of life in individuals with chronic pain. These insights are essential to healthcare policy and practice and highlight the need for a patient-centred approach to pain management that goes beyond pharmacological interventions. Integrating mental health services, expanding access to multimodal therapies, and addressing social determinants of health are essential for improving outcomes in primary healthcare contexts, particularly in low-resource environments.

### Future research

Future research should be conducted across multiple primary healthcare facilities to improve generalisability and inform broader policy development. Longitudinal studies are necessary to evaluate the progression of chronic pain and the efficacy of various treatment approaches. Incorporating standardised mental health assessment tools, such as PHQ-9 for depression (following screening with PHQ-2) and GAD-7 for anxiety, would provide a more comprehensive understanding of the psychological burden associated with chronic pain. Qualitative studies exploring patients’ lived experiences within primary healthcare could also yield valuable insights into barriers and facilitators of effective management. Finally, given the bimodal peak of pain burden observed among individuals younger than 30 and older than 75, future research should further investigate chronic pain in younger populations.

## Conclusion

This study highlights the substantial burden of chronic pain in primary healthcare settings, characterised by moderate-to-severe pain, impaired quality of life, and inadequate pain relief. These findings underscore the urgent need for more effective pain management strategies in primary healthcare clinics. Integrating pharmacological approaches, including disease-specific medications and adjuvant therapies, with non-pharmacological interventions such as physiotherapy and psychological support may enhance patient outcomes.

Primary healthcare providers should incorporate mental health support and pain education into routine care while advocating for policy reforms that expand access to multidisciplinary pain services. Policy interventions should prioritise adequate resource allocation, strengthen healthcare provider training in multimodal pain management, and promote the integration of mental health services into pain management strategies. Furthermore, addressing the key socioeconomic determinants, particularly employment status and social support, will be critical to reducing the impact of chronic pain on quality of life.
